# Modification of the Association of B-Type Natriuretic Peptides With Mortality and Hospitalization Outcomes by Sex

**DOI:** 10.1016/j.jacadv.2025.101999

**Published:** 2025-07-24

**Authors:** David Bobrowski, Husam Abdel-Qadir, Candace D. McNaughton, Anna Chu, Xuesong Wang, Peter C. Austin, Barbara S. Doumouras, Karem Abdul-Samad, Peter A. Kavsak, Michael E. Farkouh, James L. Januzzi, Heather J. Ross, Douglas S. Lee

**Affiliations:** a Department of Medicine, University of Toronto, Toronto, Ontario, Canada; b Women's College Hospital, Toronto, Ontario, Canada; c Peter Munk Cardiac Centre, University Health Network, Toronto, Ontario, Canada; d ICES (formerly known as the Institute for Clinical Evaluative Sciences), Toronto, Ontario, Canada; e Institute of Health Policy, Management, and Evaluation, University of Toronto, Toronto, Ontario, Canada; f Sunnybrook Health Sciences Centre, Toronto, Ontario, Canada; g Department of Pathology and Molecular Medicine, McMaster University, Hamilton, Ontario, Canada; h Cedars-Sinai Medical Center, Los Angeles, California, USA; i Massachusetts General Hospital, Harvard Medical School, Baim Institute for Clinical Research, Boston, Mass, USA; j Ted Rogers Centre for Heart Research, Toronto, Ontario, Canada; k Institute of Medical Science, University of Toronto, Toronto, Ontario, Canada

**Keywords:** B-type natriuretic peptide, heart failure, hospitalization, mortality, natriuretic peptides, sex

## Abstract

**Background:**

The effects of sex on the prognostic implications of natriuretic peptide (NP) elevation have not been fully elucidated in the population.

**Objectives:**

The purpose of this study was to examine if sex modifies associations of NPs with mortality and hospitalization.

**Methods:**

In a population-based retrospective cohort study, we identified all patients (aged ≥40 years) undergoing NP testing in Ontario, Canada (2015-2020). We examined for the presence of sex-by-NP interactions for 1-year outcomes and conducted sex-specific analyses for continuously increasing NP concentrations.

**Results:**

We studied 91,017 individuals with B-type natriuretic peptide (BNP) tests (median 75 years; 48.0% females) and 81,578 individuals with N-terminal pro-BNP (NT-proBNP) tests (74 years; 48.6% females). Adjusted 1-year risks of all-cause mortality at any given NP concentration were higher in males than females. For example, 1-year mortality at a BNP of 400 ng/L was 16.8% in females and 21.6% in males. At an NT-proBNP of 900 ng/L, 1-year mortality was 14.2% in females and 18.5% in males. However, there were also significant sex interactions with BNP (*P* = 0.002) and NT-proBNP (*P* = 0.03) for mortality outcomes. When we examined cardiovascular hospitalizations, there was also a significant sex-by-NP interaction. For BNP, the risk of cardiovascular hospitalization was higher in males at lower concentrations but was higher in females at higher concentrations (*P*-interaction = 0.005). For NT-proBNP, the risk of cardiovascular hospitalization was higher in males at lower NP concentrations, but the gap narrowed at higher NP levels (*P* interaction = 0.03).

**Conclusions:**

Sex modifies the association between NP concentrations and all-cause mortality or cardiovascular hospitalizations. Prognostically, interpretation of NP levels should consider effect modification by sex.

B-type natriuretic peptide (BNP) and its amino-terminal fragment, N-terminal pro-BNP (NT-proBNP), are secreted into the circulation by cardiomyocytes in response to elevated end-diastolic wall stress from ventricular volume or pressure overload.^1^ In patients with an established diagnosis of heart failure (HF), assessment of natriuretic peptide (NP) levels is recommended for prognostic risk stratification.^2^, ^3^, ^4^ NPs have also been used as measures of subclinical cardiac dysfunction for risk stratification to implement targeted HF management intervention for prevention of disease progression.^3^^,^^5^, ^6^, ^7^ Emerging reports have also demonstrated the value of NPs as risk predictors in patients with primary noncardiac pathology impacting hemodynamic status.^6^^,^^8^ We have recently shown that the use of NPs has been increasing over time.^9^ As the clinical use and potential applications of NPs continue to expand, it is important to determine whether, and to what degree, test performance may vary in different patient populations.

Many risk prediction models in cardiovascular (CV) disease such as the Framingham and the pooled cohort equations have been stratified by sex,^10^^,^^11^ and a similar sex interaction exists for stroke risk among people with atrial fibrillation.^12^^,^^13^ The extent to which the prognostic value of NPs may vary by sex in contemporary clinical practice is unclear.^14^, ^15^, ^16^, ^17^ In the general population, female sex is associated with higher circulating NP concentrations, which may be due in part, to the inhibition of NP production by testosterone.^18^ However, the absolute difference in NT-proBNP levels was modest, estimated at approximately 10 ng/L.^18^^,^^19^ Among individuals with diagnosed HF, NP levels appear to be similar or minimally higher in males, presumably due to the predominance of HF with reduced ejection fraction in males.^20^

Guidelines have suggested that when NPs are ordered as an initial test, elevated levels of this biomarker should prompt referral for echocardiography^3^ and referral to a cardiac specialist.^21^ The degree of elevation of NPs has also been suggested to have prognostic importance in the population.^22^ However, the effects of sex on the prognostic implications of BNP and NTproBNP elevation have not been fully elucidated for mortality and hospitalization outcomes in the population. Specifically, risk may differ at the same absolute NP concentration according to sex.^20^

We conducted a population-based cohort study to determine if the associations of NP concentrations with outcomes differ by sex in people with and without HF. We hypothesized that there is effect modification by sex, with higher risk of adverse outcomes in males compared to females at a given NP level in those with and without HF.

## Methods

### Data sources

Ontario is Canada's most populous province with 14.7 million residents and a single-payer, universal health care system called the Ontario Health Insurance Plan (OHIP) that pays for all medically necessary physician and hospital services. NP values, and other laboratory tests covered by OHIP, are recorded in the Ontario Laboratories Information System.^23^ The Registered Persons Database was used to obtain demographic and vital statistics information, including date of death. Hospital admissions, comorbidities, and in-hospital procedures were ascertained using the Canadian Institute for Health Information Discharge Abstract Database (which records up to 25 diagnoses, coded using the International Classification of Diseases-10th Revision) and the Same-Day Surgery database. The Ontario Drug Benefit formulary was used to determine if patients were prescribed sacubitril/valsartan in those ≥65 years of age. Data on emergency department and hospital-based ambulatory care were sourced from the National Ambulatory Care Reporting System (which records up to 10 International Classification of Diseases-10th Revision diagnoses per visit). These data sets were linked using unique encoded identifiers and analyzed at ICES (formerly the Institute for Clinical Evaluative Sciences).^24^

### Study cohort

We conducted a population-based cohort study of community-dwelling adults 40 to 105 years of age in Ontario, Canada, with at least one BNP or NT-proBNP test result between January 1, 2015, and June 30, 2020, through linkage of administrative data holdings at ICES. The study timeline was limited to the latest date of June 2020, when health services were limited due to the COVID-19 pandemic.^25^^,^^26^ We created 2 cohorts based on the first available BNP or NT-proBNP value (ie, a patient could be included in both cohorts). The study accrual period was selected for expected stability in patterns of NP testing to reduce potential biases associated with early or late adopters.^9^ Patients with a history of HF prior to the index NP test were identified using previously validated algorithms, and a lower age limit of 40 years was employed in these algorithms based on the relative rarity of HF at younger ages at the population level.^27^^,^^28^ Where an individual had multiple BNP or NT-proBNP tests, we used the initial test. We excluded individuals with missing key data (age, sex, health insurance number), nonresidents, those who were >105 years of age and residents of long-term care homes at the time of index NP testing. Since dialysis can impact NP levels, individuals with a history of dialysis in the 5 years prior to index NP were also excluded.^29^ We looked back 5 years before the index NP test to obtain baseline sociodemographic characteristics and clinical history, 3 years for laboratory values, and 6 months for medication dispensation records for those over the age of 65 years. Full details of databases used for cohort construction and variable definitions are included in Supplemental Figure 1 and Supplemental Table 1.

This study complies with the Declaration of Helsinki and was authorized under section 45 of Ontario's Personal Health Information Protection Act, which does not require review by a research ethics board. Data sharing agreements prohibit ICES from making the data set publicly available, but access may be granted to those who meet prespecified criteria for confidential access.

### Exposure

The primary exposure was the first NP concentration during the study period analyzed as a continuous variable. Since BNP and NT-proBNP values are not interchangeable and the choice of the test is made at the organizational level, analyses were stratified by BNP and NT-proBNP.^17^^,^^30^^,^^31^

### Outcomes

Study outcomes were time to all-cause mortality, CV mortality, CV hospitalization, and HF hospitalization, as previously defined.^32^ All-cause mortality was identified from the Registered Persons Database, CV mortality from the Office of the Registrar General Vital Statistics database, and HF hospitalizations from the Canadian Institute for Health Information Discharge Abstract Database.^33^ Subjects were censored after 1 year of follow-up if still event-free. Preplanned secondary analyses further stratified sex-specific outcomes by prior diagnosis of HF.

### Statistical analyses

Continuous variables were summarized using the median (25th, 75th percentile) or mean (SD), and categorical data were described using percentages. Due to the large size of the study cohort, the magnitude of differences between sexes was compared using standardized differences. Based on previous evidence of their association with NPs and outcomes,^34^ models were adjusted for history of HF, demographic characteristics (age, rural residence, and material deprivation), comorbid conditions (atrial fibrillation, diabetes, hypertension, myocardial infarction, stroke, transient ischemic attack, peripheral vascular disease, valvular heart disease, chronic obstructive lung disease, obesity, liver dysfunction, cancer, excessive alcohol use, and frailty), use of sacubitril/valsartan at baseline, laboratory values (hemoglobin and estimated glomerular filtration rate), as well as location of index NP test (emergency department, in-hospital, or outpatient).

Adjusted models using NP concentrations expressed as continuous measures centered on their mean value included an interaction term between sex and NP concentration to determine if the association between NP level and outcomes differed between males and females. NP was modeled as a continuous variable assuming a linear relationship with the log-hazard of the outcome. Since a significant interaction was observed (using a threshold of *P* < 0.05), we developed models including an interaction term for sex and NP level for all outcomes. To estimate the 1-year risk of outcomes, we used Cox regression for all-cause death and Fine-Gray regression for models of CV death and HF hospitalization, accounting for the competing risk of non-CV death and all-cause death, respectively. For illustrative purposes, we then reported the predicted probability of each outcome at 1 year at the NP value threshold over which HF is deemed “very likely” as per Cardiovascular Society guidelines (ie, BNP of 400 ng/L or NT-proBNP of 900 ng/L for ages 50-75 years).^3^ We obtained risks at particular NP concentrations using reference female and male patients at the overall mean age with no comorbidities and a Hospital Frailty Risk Score^35^ <5 who were assigned to the lowest deprivation quintile and living in an urban area. In planned secondary analyses, we stratified by any history of HF. All analyses were performed using SAS Enterprise Guide 7.1 (SAS Institute Inc). A 2-sided *P* value <0.05 was considered statistically significant.

## Results

### Baseline characteristics

After applying the exclusion criteria (Supplemental Figure 1), the BNP cohort included 91,017 individuals (48.0% females), and the NT-proBNP cohort included 81,578 individuals (48.6% females). Baseline characteristics of each NP cohort stratified by sex are reported in Table 1. The median age of individuals in the BNP cohort was 75 years (25th, 75th percentiles: 65, 84) and 74 years (65, 83) in the NT-proBNP cohort. The median BNP and NT-proBNP plasma concentrations were 189 ng/L (59, 537) and 955 ng/L (198, 3,720), respectively. There were 38,402 (42.2%) people in the BNP cohort and 36,736 (45.0%) in the NT-proBNP cohort with prior HF. People with documented pre-existing HF had higher median NP levels (BNP 389 ng/L [155, 906]; NT-proBNP 2,473 ng/L [793, 6,786]) than people without documented pre-existing HF (BNP 103 [36, 296]; NT-proBNP 345 ng/L [98, 1,546]). BNP was only mildly lower in obese vs nonobese individuals (161 [59, 381] vs 190 [59, 543]) as was NTproBNP (842 [209, 2,773] vs 959 [197, 3,760]) in the population. Most NP tests were performed in the acute care setting (in-hospital or emergency department). This was more prominent for BNP (70.1% of tests) than NT-proBNP (66.7% of tests). Between 2015 and 2018, a greater proportion of index NP tests were BNP (57.1% of tests) than NT-proBNP (42.9% of tests), with increased utilization of NT-proBNP (53.5% of tests) from 2019 to 2020.

**Table 1:** Baseline Characteristics at the Time of First NP Test, Stratified by NP Test Type

	BNP Cohort			NT-proBNP Cohort		
	Male (n = 47,297, 52.0%)	Female (n = 43,720, 48.0%)	Std diff	Male (n = 41,907, 51.4%)	Female (n = 39,671, 48.6%)	Std diff
Age, y	73 (64, 82)	77 (66, 85)	0.20	73 (64, 82)	75 (66-84)	0.17
Absolute NP concentration, ng/L	195 (59, 564)	183 (59, 512)	0.03	1,049 (214, 3,919)	856 (184, 3,529)	0.06
NP test location						
Emergency department	20,942 (44.3%)	20,384 (46.6%)	0.05	18,010 (43.0%)	17,569 (44.3%)	0.03
In-hospital	11,723 (24.8%)	10,792 (24.7%)	<0.01	9,757 (23.3%)	9,037 (22.8%)	0.01
Outpatient	14,632 (30.9%)	12,544 (28.7%)	0.05	14,140 (33.7%)	13,065 (32.9%)	0.02
Material deprivation quintile						
1 (least deprivation)	9,690 (20.5%)	8,150 (18.6%)	0.05	8,343 (19.9%)	7,444 (18.8%)	0.03
2	9,150 (18.6%)	8,171 (18.7%)	0.02	7,850 (18.7%)	7,083 (17.9%)	0.02
3	8,549 (18.1%)	7,913 (18.1%)	<0.01	8,009 (19.1%)	7,426 (18.7%)	0.01
4	9,616 (20.3%)	9,293 (21.3%)	0.02	8,370 (20.0%)	8,183 (20.6%)	0.02
5 (greatest deprivation)	9,944 (21.0%)	9,905 (22.7%)	0.04	8,806 (21.0%)	9,119 (23.0%)	0.05
Rural residence	3,990 (8.4%)	2,981 (6.8%)	0.06	4,704 (11.2%)	3,770 (9.5%)	0.06
Diagnosed heart failure	20,615 (43.6%)	17,787 (40.7%)	0.06	19,634 (46.9%)	17,102 (43.1%)	0.08
Ischemic heart disease	23,976 (50.7%)	16,949 (38.8%)	0.24	21,379 (51.0%)	15,140 (38.2%)	0.26
Myocardial infarction	4,965 (10.5%)	2,983 (6.8%)	0.131	4,853 (11.6%)	3,147 (7.9%)	0.12
Peripheral vascular disease	2,269 (4.8%)	1,190 (2.7%)	0.11	2,006 (4.8%)	1,075 (2.7%)	0.11
Stroke	1,759 (3.7%)	1,576 (3.6%)	0.01	1,748 (4.2%)	1,464 (3.7%)	0.03
Atrial fibrillation	14,837 (31.4%)	12,206 (27.9%)	0.08	13,543 (32.3%)	10,955 (27.6%)	0.10
Valvular heart disease	3,464 (7.3%)	2,847 (6.5%)	0.03	2,979 (7.1%)	2,575 (6.5%)	0.03
Diabetes	20,868 (44.1%)	17,373 (39.7%)	0.09	19,177 (45.8%)	15,742 (39.7%)	0.12
Hypertension	37,638 (79.6%)	35,387 (80.9%)	0.03	33,491 (79.9%)	31,746 (80.0%)	0.71
Obesity	1,385 (2.9%)	1,527 (3.5%)	0.032	1,294 (3.1%)	1,641 (4.1%)	0.056
Emphysema	17,792 (37.6%)	15,082 (34.5%)	0.07	15,997 (38.2%)	14,380 (36.2%)	0.04
Liver dysfunction	1,379 (2.9%)	822 (1.9%)	0.07	1,147 (2.7%)	692 (1.7%)	0.07
Alcohol use disorder	2,585 (5.5%)	832 (1.9%)	0.19	2,245 (5.4%)	759 (1.9%)	0.19
Cancer	13,583 (28.7%)	11,525 (26.4%)	0.05	10,690 (25.5%)	9,804 (24.7%)	0.02
Frailty risk score (HFRS)	4 (1, 8)	4 (1, 9)	0.06	4 (1, 9)	4 (1, 10)	0.07
Sacubitril/valsartan use^a^	508 (1.1%)	153 (0.3%)	0.086	569 (1.4%)	199 (0.5%)	0.089
Serum sodium, mEq/L	139 (136, 141)	139 (136, 141)	0.01	139 (136, 141)	139 (136, 141)	0.02
Serum hemoglobin, g/L	129 (110, 144)	121 (106, 134)	0.35	130 (111, 144)	122 (106, 134)	0.36
eGFR, mL/min/1.73 m^2^	70 (48, 89)	67 (46, 87)	0.08	71 (49, 90)	69 (47, 88)	0.05

BNP = B-type natriuretic peptide; eGFR = estimated glomerular filtration rate; HFRS = Hospital Frailty Risk Score; NP = natriuretic peptide; NT-proBNP = N-terminal pro-B-type natriuretic peptide; Std diff = standardized difference.

a Sacubitril/valsartan use determined only in those ≥65 years of age in ODB formulary.

The BNP cohort included 17,787 females (40.7%) and 20,615 males (43.6%) with a prior diagnosis of HF, while the NT-proBNP cohort consisted of 17,102 females (43.1%) and 19,634 males (46.9%) previously diagnosed with HF. Compared to males, females had lower BNP and NT-proBNP concentrations, were older, and had fewer pre-existing conditions including diagnosed CV disease (ischemic heart disease, atrial fibrillation, peripheral vascular disease, valvular heart disease), diabetes, and chronic kidney disease. Females were also less likely to have non-CV comorbidities, such as liver dysfunction, cancer, or chronic obstructive lung disease. Females in the BNP cohort had a higher prevalence of hypertension, while there was no sex difference in hypertension within the NT-proBNP cohort.

### Sex-specific mortality outcomes by natriuretic peptide concentration

**Figure 1:**
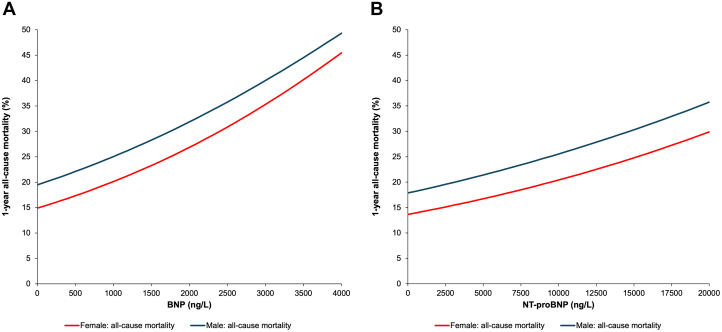
Risk of All-Cause Mortality by NP Concentration, Stratified by Sex (A) Adjusted 1-year risk of all-cause mortality by BNP concentration, stratified by sex. Probability of 1-year all-cause mortality was higher in males (blue line, top) than females (red line, bottom). 95% CIs are plotted but are too narrow to be visible on figure. The sex-by-BNP interaction for the hazard of all-cause death was statistically significant (*P* = 0.002). (B) Adjusted 1-year risk of all-cause mortality by NT-proBNP concentration, stratified by sex. Probability of 1-year all-cause mortality was higher in males (blue line, top) than females (red line, bottom). 95% CIs are plotted but are too narrow to be visible on figure. The sex-by-NT-proBNP interaction for the hazard of all-cause death was statistically significant (*P* = 0.025). BNP = B-type natriuretic peptide; NT-proBNP = N-terminal pro-B-type natriuretic peptide.

**Figure 2:**
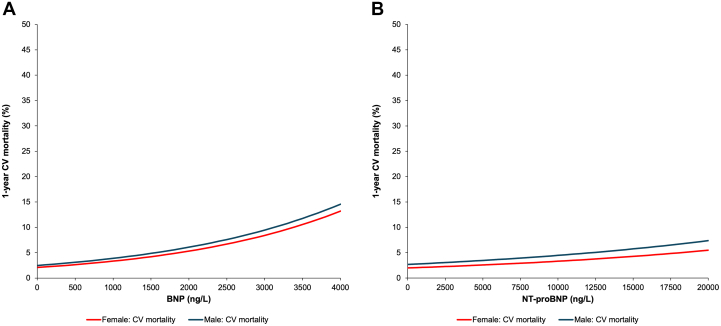
Risk of CV Death by NP Concentration, Stratified by Sex (A) Adjusted 1-year risk of cardiovascular death by BNP concentration, stratified by sex. The adjusted cumulative incidence of cardiovascular death was comparable in males (blue line) and females (red line), with no sex-by-BNP interaction for cardiovascular death (*P* = 0.53). 95% CIs are plotted but are too narrow to be visible on figure. (B) Adjusted 1-year risk of cardiovascular death by NT-proBNP concentration, stratified by sex. The adjusted cumulative incidence of cardiovascular death was comparable in males (blue line) and females (red line), with no sex-by-NT-proBNP interaction for cardiovascular death (*P* = 0.97). 95% CIs are plotted, but are too narrow to be visible on figure. CV = cardiovascular; other abbreviations as in Figure 1.

One-year follow-up status was known for 90,892 (99.9%) of those who underwent BNP testing and 81,466 (99.9%) undergoing NT-proBNP tests. Lack of vital status information, due to loss of OHIP eligibility, was similar: 0.1% vs 0.1% for BNP and 0.1% vs 0.2% for NT-proBNP, in males and females, respectively. Using a Cox model, the adjusted risk of 1-year all-cause mortality increased in a linear fashion as NP levels increased. A significant sex-based interaction for the association between NP concentration and the hazard of all-cause death (*P* = 0.002 for BNP; *P* = 0.025 for NT-proBNP) was also observed (Table 2). The 1-year probability of all-cause mortality at any given NP concentration was higher in males compared to females after adjusting for demographic characteristics and comorbidities (Figures 1A and 1B). For example, BNP concentrations of 400 ng/L corresponded to an adjusted risk of 1-year mortality of 16.2% (95% CI: 15.2%-17.2%) for females vs 20.8% (95% CI: 19.5%-22.1%) in males (Supplemental Table 2). At NT-proBNP concentrations of 2,000 ng/L, the adjusted risk of 1-year mortality was 15.2% (13.7, 16.6%) for females vs 19.7% (17.9, 21.5%) in males. Using a Fine-Gray model, the adjusted cumulative incidence of CV death (Figures 2A and 2B) was comparable between males and females across the range of NP concentrations, with no significant sex interaction (both *P* > 0.50) (Table 2).

**Table 2:** *P* Values for Testing the Interaction Between Sex and NP Concentration From Clinical Outcome Models

	*P* Value for Interaction Between Sex and NP		
	BNP		
Outcome of BNP Cohort	Overall	No Heart Failure	Heart Failure
All-cause death	0.002	0.004	0.116
Cardiovascular death	0.53	0.36	0.94
Heart failure hospitalization	0.68	0.28	0.84
CV hospitalization	<0.001	0.532	0.005

	**NT-proBNP**		
**Outcome of NT-proBNP Cohort**	**Overall**	**No Heart Failure**	**Heart Failure**

All-cause death	0.025	0.171	0.300
Cardiovascular death	0.97	0.49	0.94
Heart failure hospitalization	0.20	0.047	0.64
CV hospitalization	0.062	0.420	0.028

CV = cardiovascular; other abbreviations as in Table 1.

### Sex-specific hospitalization outcomes by natriuretic peptide concentration

**Figure 3:**
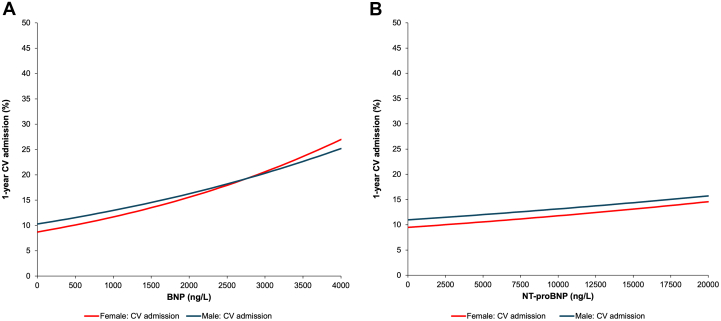
Risk of CV Hospitalization by NP Concentration, Stratified by Sex (A) Adjusted 1-year risk of cardiovascular hospitalization by BNP concentration, stratified by sex. The adjusted cumulative incidence of cardiovascular hospitalization for males (blue line) and females (red line) exhibited a significant sex-by-BNP interaction (*P* < 0.001). 95% CIs are plotted, but are too narrow to be visible on figure. At high BNP levels, risk of cardiovascular hospitalization was higher in females than males. (B) Adjusted 1-year risk of cardiovascular hospitalization by NT-proBNP concentration, stratified by sex. The adjusted cumulative incidence of cardiovascular hospitalization for males (blue line) and females (red line) exhibited a trend toward a sex-by-BNP interaction (*P* = 0.062). 95% CIs are plotted, but are too narrow to be visible on figure. Risk of cardiovascular hospitalization was higher in males than females. Abbreviations as in Figures 1 and 2.

**Figure 4:**
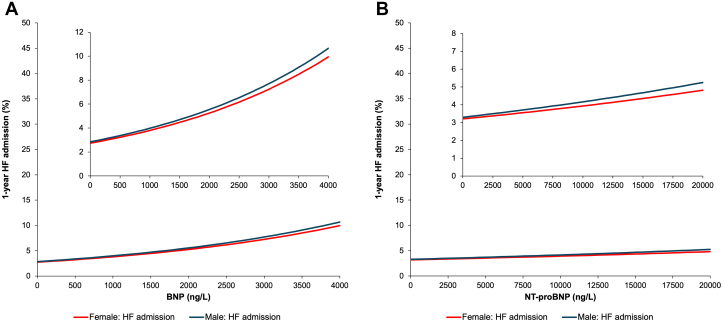
Risk of Heart Failure Hospitalization by NP Concentration, Stratified by Sex (A) Adjusted 1-year risk of heart failure hospitalization by BNP concentration, stratified by sex. Sex-by-BNP interaction for heart failure hospitalization was not significant (*P* = 0.68). 95% CIs are plotted, but are too narrow to be visible on figure. (B) Adjusted 1-year risk of heart failure hospitalization by NT-proBNP concentration, stratified by sex. Sex-by-NT-proBNP interaction for heart failure hospitalization was not significant (*P* = 0.20). 95% CIs are plotted, but are too narrow to be visible on figure. HF = heart failure; other abbreviations as in Figure 1.

The Fine-Gray models for adjusted cumulative incidence of CV hospitalization demonstrated a significant interaction by sex for BNP (*P* < 0.001) but there was a borderline sex interaction for NT-proBNP for this outcome (*P* = 0.062). A significant sex interaction could be observed visually for BNP as the CV hospitalization curves crossed between 2,500 and 3,100 ng/L (Figure 3A). For NT-proBNP, the difference in the CV hospitalization curves narrowed as NT-proBNP approached extremely high concentrations (Figure 3B). The Fine-Gray models for adjusted 1-year risk of HF hospitalization demonstrated no significant interactions by sex with interaction *P* values ≥0.2 for both BNP and NT-proBNP (Table 2). The average rate of increase of 1-year adjusted risk of HF hospitalization events for any given increase in NP concentration was lower for HF-specific hospitalizations compared to all other outcomes examined (Figure 4A). This attenuation of the “slope” of this relationship was most pronounced for NT-proBNP (Figure 4B).

### Stratified analyses based on prior HF

Patterns of association of NP concentrations with outcomes stratified by prior HF status are shown in Table 2. When stratified by prior HF status, the sex interaction of BNP for all-cause death was highly significant in those without prior HF (*P* = 0.004). Conversely, when stratified by prior HF status, the interaction of BNP with sex for the outcome of CV hospitalization was highly significant in those prior HF (*P* = 0.005). For the same CV hospitalization outcome, there was also a significant interaction of sex and NT-proBNP levels in those with prior HF (*P* = 0.028).

## Discussion

This population-based cohort of more than 90,000 people with BNP and more than 80,000 people with NT-proBNP results between 2015 and 2020 examined whether sex modified the association between NP levels and the risk of all-cause mortality, CV hospitalization, or HF hospitalization. The adjusted risk of all-cause mortality was higher for males compared to females across BNP and NT-proBNP concentrations, and there was a significant sex interaction for both NPs (Central Illustration). Interestingly, the sex interaction was most significant for BNP in those without prior HF. There were significant sex interactions for the outcome of CV hospitalization, which were driven primarily by those with prior HF. Our study suggests that the impact of NPs is complex and is dependent in part by sex, prior HF status, and the specific outcome of interest.

**Central Illustration:**
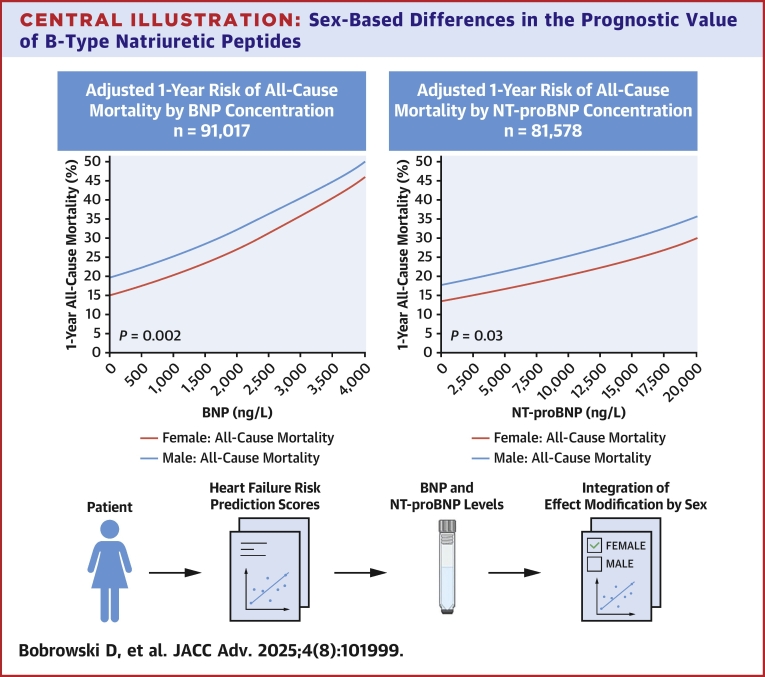
Sex-Based Differences in the Prognostic Value of B-Type Natriuretic Peptides Adjusted 1-year risk of all-cause mortality by B-type natriuretic peptide (BNP, left) and N-terminal pro-BNP (NT-proBNP, right) concentrations demonstrates a sex-based difference, with higher mortality risk observed in males compared to females at any given peptide level. Significant interactions by sex were noted for both BNP (*P* = 0.002) and NT-proBNP (*P* = 0.03), indicating that sex modifies the association between natriuretic peptide levels and mortality. 95% CIs are plotted but are too narrow to be visible on figure. These findings demonstrate the importance of integrating sex-specific interpretation into heart failure risk prediction models that incorporate natriuretic peptide biomarkers.

An analysis of the Essen Coronary Artery Disease registry including patients without HF found that males have a lower threshold for NPs than females for predicting all-cause mortality, a finding consistent with our results.^36^ Our findings add to growing evidence that the predictive value of NP differs by sex. The Korean Heart Failure Registry found that NT-proBNP was independently associated with all-cause mortality in males but not females after discharge from hospital.^16^ A trial among 567 patients with HF demonstrated an interaction between sex and mortality, with higher 3-year all-cause mortality in males compared to females, particularly at lower levels of BNP.^37^ Another prospective cohort study of unselected elderly patients in the primary care setting found that a 1-U change in log-transformed NT-proBNP increased the hazard of all-cause mortality by 38.9% in females and 26.7% in males (*P* interaction = 0.02).^38^ Our population-based analysis examines interactions between sex and NP levels in a large, general population sample. The convergence between our study and prior research emphasizes that sex-specific models using NP levels may yield higher predictive accuracy for all-cause mortality and CV hospitalizations, compared to models that do not consider sex. At the Canadian Cardiovascular Society guideline threshold designating HF as “very likely” (ie, BNP of 400 ng/L or NT-proBNP of 900 ng/L for ages 50-75 years), the absolute adjusted 1-year all-cause mortality was 4.9% and 3.3% higher, respectively, in males compared to females.^3^

Some prior studies showed no sex-related differences in NPs and outcomes. For example, a substudy of the GUIDE-IT (Guiding Evidence Based Therapy Using Biomarker Intensified Treatment in Heart Failure) trial found no sex differences in HF hospitalization, CV hospitalization, or composite outcome of CV death and HF hospitalization at 1- and 2-year follow-up after adjustment for baseline characteristics, albeit in the context of a randomized controlled trial and with highly detailed information regarding extent of HF risk factors and knowledge of left ventricular ejection fraction (LVEF).^14^ Furthermore, among elderly primary care patients, there was also no significant interaction between sex and continuous NT-proBNP values for CV mortality.^38^ However, these studies were much smaller than our analysis, with sample sizes of <1,000 patients in the GUIDE-IT substudy,^14^ and <6,500 patients with no analysis of CV hospitalizations in the latter study.^38^ In contrast, our study examined a range of outcomes in a population-based setting examining over 170,000 NP tests.

The reasons for divergence in the relationship between NP levels and outcomes according to sex warrant further investigation. NPs had more pronounced effect on the broader category of all-cause mortality than the more specific outcome of CV death. Similarly, NPs had more pronounced effects on the broader category of CV hospitalizations than HF-specific hospitalizations. Elevated circulating NP levels among patients may reflect CV, as well as non-CV hemodynamic stressors, such as cirrhosis or pulmonary embolism. Accordingly, sex-specific comorbidities may mediate the association of NP concentrations with outcomes in females and males.^2^^,^^6^^,^^8^ Females in our cohort were older, but had fewer diagnosed comorbidities than males, which may be related to differences in biology, social determinants of health, underlying etiology of HF, or access to medical care for diagnostic testing. Coronary artery disease and myocardial infarction are leading causes of HF in males.^20^^,^^39^ The lower prevalence of chronic obstructive pulmonary disease and liver dysfunction is in keeping with lower rates of smoking and alcohol use in females.^14^^,^^40^ Differences in NP concentration may also relate to prevalence of HF with reduced vs preserved ejection fraction in males and females.^20^^,^^37^^,^^39^ In a secondary analysis of a multicenter clinical trial, absolute NT-proBNP values were consistently lower in females with HF with reduced ejection fraction, while the 1-year rate of change in NT-proBNP was similar between sexes.^14^ The effect of circulating sex steroid hormones exhibit minor differences in NP concentrations.^19^^,^^41^ An inverse association exists between testosterone and NP levels, while estrogen therapy increases NP concentrations.^30^^,^^42^, ^43^, ^44^, ^45^ Free hormone levels may modulate NP concentrations, as sex hormone-binding globulin levels are positively associated with NT-proBNP in both sexes.^30^^,^^42^^,^^43^ In males, sex hormone-binding globulin concentrations increase with age, while in females, they rise after menopause. In our study, the higher prevalence of pre-existing HF and CV disease in males, compared to postmenopausal females, may better explain larger differences in NP concentrations.

Guidelines recommend the integration of validated multivariable risk scores into clinical practice to predict the development of incident HF and outcomes in ambulatory or hospitalized patients.^3^^,^^4^ However, sex is often not included in predictive models that incorporate NPs in the model. For example, the CODE-HF decision tool which was designed to improve the diagnosis of HF using multiple covariate adjustment did not incorporate sex in their models, which may have been due, in part, to lack of statistical association.^46^ From the standpoint of prognosis, some predictive models incorporate NPs but do not currently account for sex, and thus it is unclear if the performance of such models would differ in males and females.^47^ Other HF risk scores, that do not incorporate NPs,^48^ have demonstrated predictiveness and utility in improving outcomes prospectively and in randomized controlled trials, without including either sex or NPs as covariates.^49^^,^^50^ However, future studies that utilize NPs in prognostic models which include all-cause death as an outcome should consider whether adjustment or stratification by sex is warranted to achieve optimal performance. Our findings suggest that prognostication using NP values for all-cause mortality or CV hospitalization outcomes should consider the importance of effect modification by sex.

Finally, our study demonstrated that risk of adverse outcomes increased progressively with rising NP values even at the lower range of BNP (<400 ng/L) or NT-proBNP (<2,000 ng/L). This association was most marked for all-cause death and CV hospitalization and suggests that there is clinically important increase in risk of HF events even at the lower range of NP values. Furthermore, these findings highlight that at NP concentrations that are considered to be “indeterminate” (ie, BNP of 100-400 ng/L or NT-proBNP of 300-900 ng/L for ages 50-75 years), clinicians should consider if more intensive investigation or treatment may be indicated as NP values vary within this range.^3^ These considerations may impact the ability to intervene and may potentially allow for prevention or delay of incident HF hospitalizations.^28^

### Study Limitations

Our study has several potential limitations. We were unable to categorize HF diagnosis according to LVEF in our cohort since such information was unavailable in our databases. However, the presence of important CV risk factors was captured in baseline characteristics, current thresholds for NP concentrations used in HF guidelines do not differentiate by LVEF,^3^, ^4^, ^5^ and prognostic models for HF do not necessitate inclusion of this covariate.^51^, ^52^, ^53^ It is possible that LVEF may explain, in part, the sex differences observed. Although we were unable to adjust for heart failure with preserved ejection fraction or heart failure with reduced ejection fraction status, we adjusted for characteristics that are associated with either phenotype such as atrial fibrillation, diabetes, hypertension, myocardial infarction, and valvular heart disease.^54^ A cohort study consisting of 2,042 patients found the discriminatory value for BNP for detection of reduced ejection fraction increased with age and was higher in females; however, only 8 female subjects had an ejection fraction of ≤40% and this significantly limited the power to demonstrate meaningful sex differences.^55^ Despite this, future studies may elucidate whether LV ejection fraction may be a mediator of the sex differences observed. It is also important to highlight that our findings apply to the difference between sexes in the application of NP levels for prognosis, rather than diagnosis. Moreover, the actual diagnostic company assays that were used to generate the NP results were not available which precluded a more specific assay-adjusted analysis besides the adjustment for the hospital wherein laboratory recommendations advocate for using only one NP assay in the hospital setting. However, the advantages of our analysis include the large population-based analysis and the array of outcomes that could be examined with complete follow-up in analyses that account for competing risks. Available data sources for this study did not contain risk factors such as body mass index or race, however, we did account for important socioeconomic factors such as material deprivation.^17^^,^^20^^,^^34^ Additionally, we studied single NP values that were not collected in a systematic manner, which may have resulted in a heterogeneous sample and selection bias. Although we adjusted for location of NP testing (hospital, emergency department, or outpatient), we lacked data on functional classification and evidence of acute decompensation. However, the advantages of our analysis include the large population-based analysis and the array of outcomes that could be examined with complete follow-up in analyses that account for competing risks.

## Conclusions

In this population-based cohort, females had a lower adjusted risk of 1-year all-cause mortality compared to males at the same BNP and NT-proBNP values, and there was evidence of effect modification by sex for the broad outcome of all-cause mortality. Sex also modified the association between NP levels and CV hospitalization. However, we did not find evidence for effect modification by sex for associations between NP values and CV death or HF hospitalization for people with or without history of HF. Prognostic studies and clinical prediction models of all-cause mortality or CV hospitalization that include NPs as variables should consider the possibility of effect modification by sex.

## Funding support and author disclosures

This study was supported by a Foundation grant from the 10.13039/501100000024Canadian Institutes of Health Research (grant # FDN 148446) and also supported by the ICES, which is funded by an annual grant from the Ontario Ministry of Health (MOH) and the Ministry of Long-Term Care (MLTC). Dr Abdel-Qadir was supported by a National New Investigator Award from the Heart and Stroke Foundation of Canada and is currently supported by Tier 2 Canada Research Chair in Cardiovascular Disease Epidemiology and Outcomes. Dr McNaughton is supported by the Sunnybrook Research Institute, the Practice Plan of the Department of Emergency Services at Sunnybrook Health Sciences Centre and the University of Toronto. Dr Lee is the Ted Rogers Chair in Heart Function Outcomes, University Health Network, University of Toronto. Dr Doumouras is supported by the Women in Cardiology Fund from the 10.13039/501100020895Temerty Faculty of Medicine, University of Toronto. Dr Kavsak has received grants/reagents/consultant/advisor/honoraria from diagnostic companies that manufacture NP assays and materials for testing, including Abbott Laboratories, Abbott Point of Care, Beckman Coulter, Ortho Clinical Diagnostics, Quidel, Randox Laboratories, Roche Diagnostics, Siemens Healthcare Diagnostics, and Thermo Fisher Scientific. Dr Januzzi reports equity holdings in Imbria Pharma, Jana Care, and Fibrosys, current/recent grant support from Abbott, Applied Therapeutics, AstraZeneca, BMS, Novartis Pharmaceuticals, consulting income from Abbott Diagnostics, Beckman-Coulter, Jana Care, Janssen, Novartis, Prevencio, Quidel, and Roche Diagnostics, and serves on clinical endpoint committees/data safety monitoring boards for Abbott, AbbVie, Amgen, CVRx, Medtronic, Pfizer, and Roche Diagnostics. All other authors have reported that they have no relationships relevant to the contents of this paper to disclose.
