# Supplementary material for: Modification of the Association of B-Type Natriuretic Peptides With Mortality and Hospitalization Outcomes by Sex
Source: JACC Adv. 2025 Jul 24;4(8):101999. doi: 10.1016/j.jacadv.2025.101999 (PMC12311523; doi:10.1016/j.jacadv.2025.101999)
Supplement: Supplementary data [file mmc1.docx]

Supplemental Figure 1: Cohort creation flow diagram


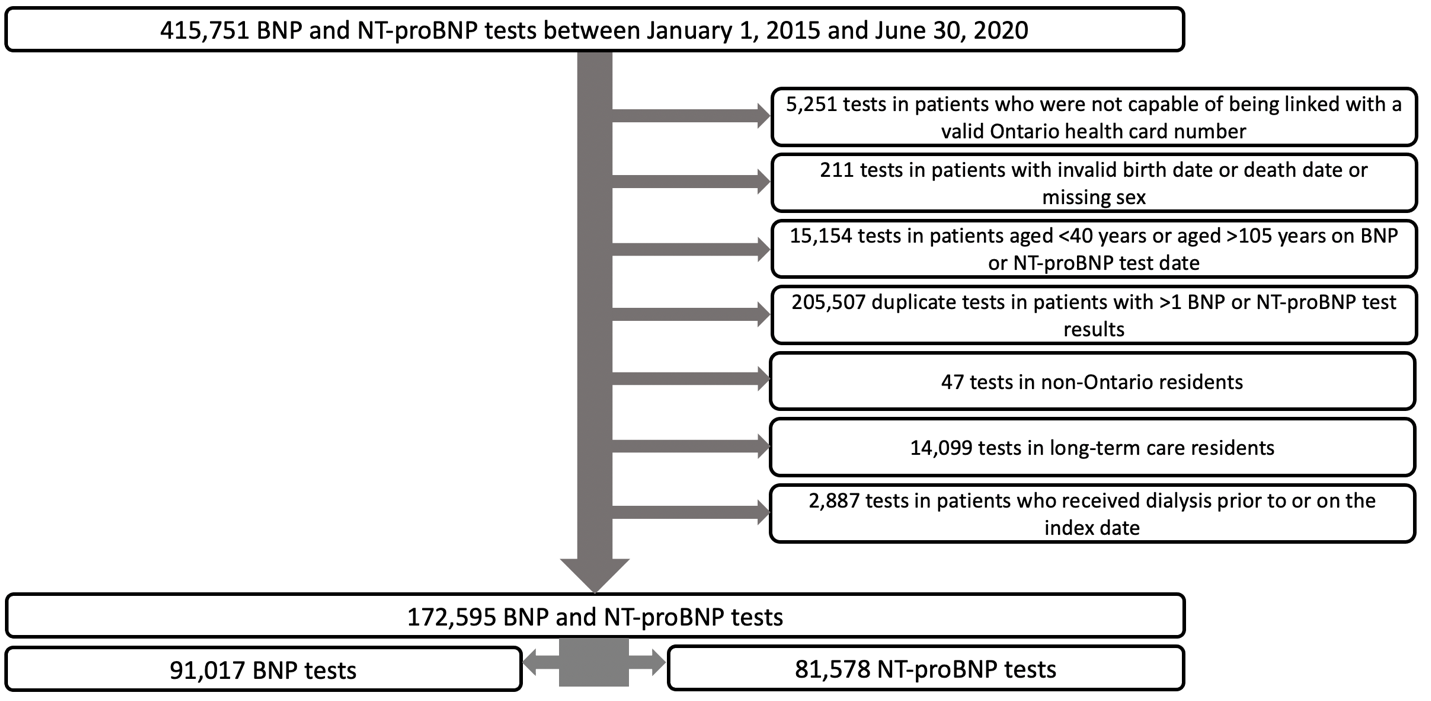


Supplemental Figure 2: Adjusted 1-year survival, stratified by diagnosed heart failure, for males and females by BNP concentration: all-cause death (panels A and B), cardiovascular death (panels C and D), and heart failure hospitalization (panels E and F)


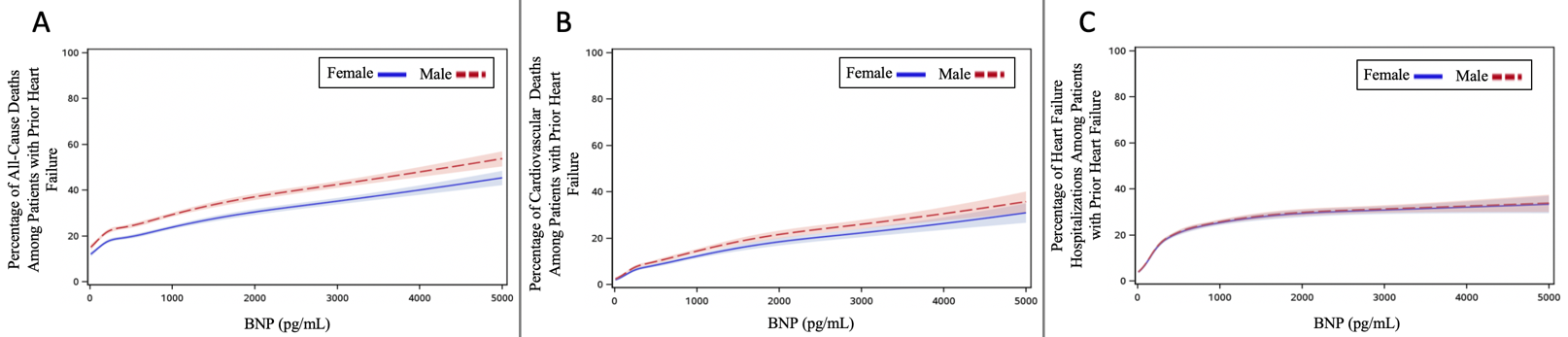

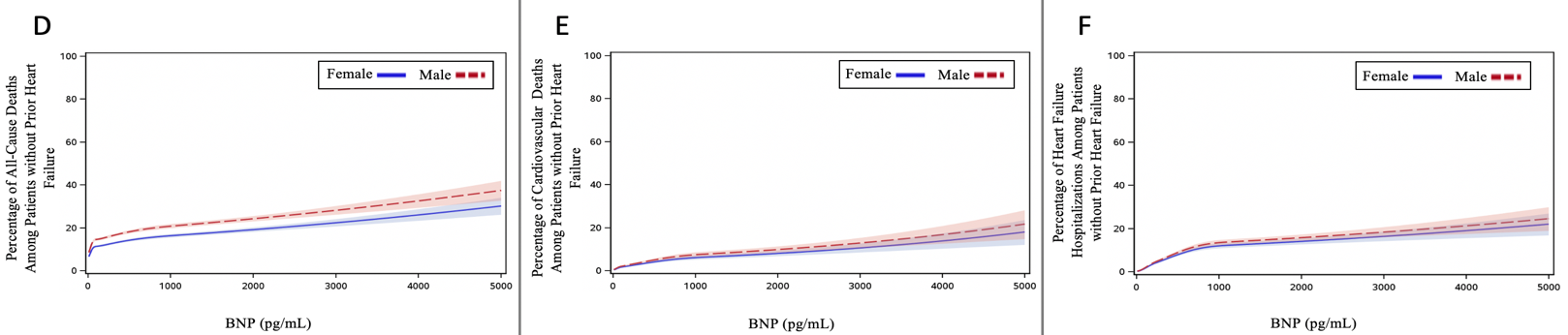

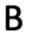

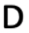

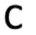

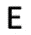


Supplemental Figure 3: Adjusted 1-year survival, stratified by diagnosed heart failure, for males and females by NT-proBNP concentration: all-cause death (panels A and B), cardiovascular death (panels C and D), and heart failure hospitalization (panels E and F)


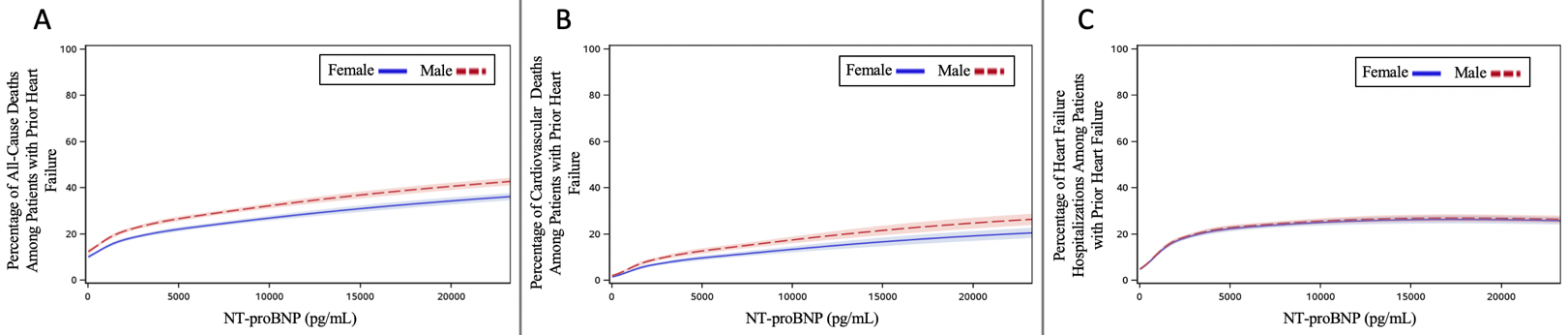

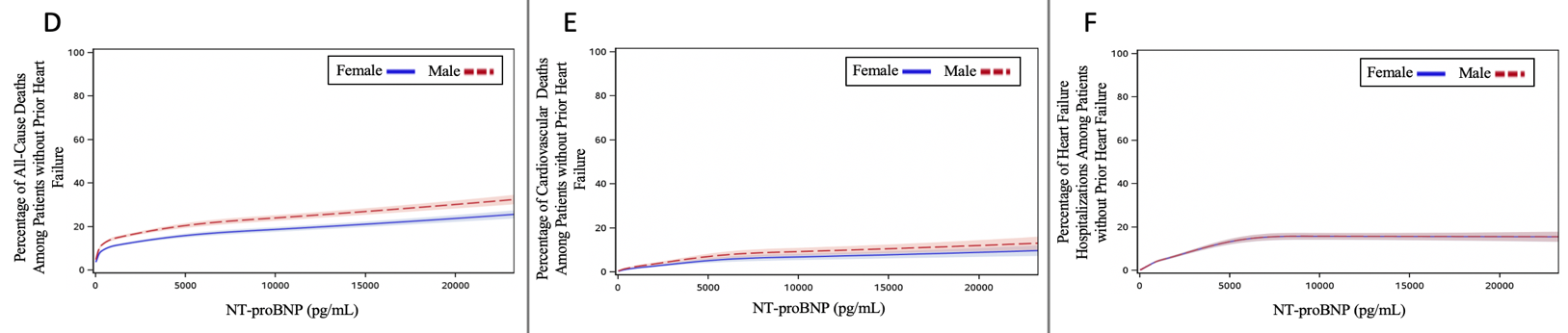

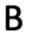

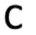

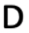

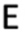


Supplemental Table 1: Definition of exposure, covariates, and outcomes

| **Exposure** | **Data Source** | **Definition** |
| --- | --- | --- |
| Natriuretic Peptide Test | Ontario Laboratories Information System | The first test result for B-type natriuretic peptide and/or N-terminal pro-brain natriuretic peptide. |
| **Covariate** | **Data Source** | **Definition** |
| Age (continuous) | Ontario Registered Persons Database | ≥ 40 or ≤ 105 years of age as of index natriuretic peptide test date. |
| Sex | Ontario Registered Persons Database | Male or Female |
| Material deprivation | Ontario Marginalization Index | Using the smallest standard geographic area for which census data are reported (400-700 persons) to determine the extent of material deprivation of the neigbourhood of a person’s residence. A composite measure that includes the proportion of:   - the population age 20+ years without a high-school diploma - families who are lone parent families - total income from government transfer payments for population age 15+ - the population age 15+ who are unemployed - the population considered low-income - households living in dwellings in need of major repair   Quintile 1 Lowest deprivation  Quintile 2 Next to lowest  Quintile 3 Middle  Quintile 4 Next to highest  Quintile 5 Highest deprivation |
| Rural residency | Postal Code Conversion File | Community size = <10,000 |
| Location of Test | Discharge Abstract Database  Same Day Surgery Database  National Ambulatory Care Reporting System  Ontario Health Insurance Plan | Location where index natriuretic peptide test was performed. Locations include emergency department, in-hospital, and outpatient settings. |
| Atrial fibrillation | Discharge Abstract Database  National Ambulatory Care Reporting System  Ontario Health Insurance Plan | Lookback 5 years from index admission date:  1 hospitalization or 1 ED visit (ICD-10-CA I48)  or 4 outpatient claims (ICD-9 427) in a one-year period |
| Chronic obstructive pulmonary disease | Discharge Abstract Database  Same Day Surgery Database  Ontario Health Insurance Plan | Lookback to 1991:  ≥1 hospitalization (ICD-9 491, 492, 496; ICD-10-CA J41, J42, J43, J44) *or* ≥3 outpatient claims (diagnosis codes 491, 492, 496) in a two-year period |
| Ischemic Heart Disease | Discharge Abstract Database  Same Day Surgery Database | Lookback 5 years for previous hospitalization for or day surgery for myocardial infarction, percutaneous coronary intervention, or coronary artery bypass graft:  ICD-10-CA I21, I22, or CCI 1IJ50, 1IJ57GQ, 1IJ54, 1IJ76 |
| Dementia | Discharge Abstract Database  Same Day Surgery Database  Ontario Health Insurance Plan  Ontario Drug Benefit Program | Lookback to 1988:  ≥1 hospital or same day surgery admission (ICD-9 46.1, 290.0x, - 290.4x, 294.1x, 294.2x, 331.0x, 331.1x, 331.5x) (ICD-10-CA F00.x – F03.x, G30.x) or  ≥1 drug claim for cholinesterase inhibitors or  ≥3 outpatient claims (ICD-9 290, 331) at least 30 days apart in a two-year period |
| Diabetes | Discharge Abstract Database  Ontario Health Insurance Plan  Ontario Drug Benefit Program | Lookback to 1991:  ≥2 outpatient claims (ICD-9 250) in a one-year period, or ≥1 hospitalization (ICD-10-CA E10, E11, E13, E14), or ≥1 diabetes drug claim in a one-year period |
| Dyslipidemia | Discharge Abstract Database  Same Day Surgery Database  Ontario Health Insurance Plan | Lookback to 1991:  1 hospitalization (ICD-9 272 or ICD-10-CA E78 as any diagnosis), or 2 outpatient claims (ICD-9 272) within 2 years, or 1 outpatient claim followed by 1 hospitalization within 2 years |
| Hypertension | Discharge Abstract Database  Same Day Surgery Database  Ontario Health Insurance Plan | Lookback to 1991:  ≥2 outpatient claims (ICD-9 401.x, 402.x, 403.x, 404.x, or 405.x) in a two-year period, or  1 outpatient + 1 hospitalization or day surgery record (ICD-10-CA I10.x, I11.x, I12.x, I13.x, or I15.x) in a two-year period, or  If no outpatient record is found, ≥1 hospitalization or day surgery record |
| Obesity | Discharge Abstract Database  Same Day Surgery Database  National Ambulatory Care Reporting System | Lookback 5 years:  ICD-10-CA E66.x |
| Peripheral vascular disease | Discharge Abstract Database  Same Day Surgery Database  National Ambulatory Care Reporting System | Lookback 5 years for previous hospitalization or day surgery for abdominal aortic aneurysm, or peripheral artery disease (any diagnosis type):  ICD-10-CA I702, I713, I714, I739, I743, I744 or  CCI 1JE50, 1JE76, 1JJ76, 1JK76, 1JM76, 1JX76, 1JY76, 1JE50, 1JJ50, 1JK50, 1JL50, 1JM50, 1JQ50, 1JT50, 1JU50, 1JW50, 1JX50, 1JY50, 1KA76, 1KE76, 1KG76, 1KQ76, 1KR76, 1KT76 1KY76, 1KA50, 1KE50, 1KG50, 1KQ50, 1KR50, 1KT50, 1KY50, 1KZ50 |
| Recent cancer | Ontario Cancer Registry | Lookback 5 years for any record |
| Dialysis | Ontario Health Insurance Plan  Canadian Organ Replacement Registry | 2 OHIP fee codes at least 90 days apart within 6 months prior to index test date: R849, G323, G325, G326, G330, G331, G332 (only before 2008), G860, G333, G083, G091, G085, G295, G082, G090, G092, G093, G094, G861 - G866, G294, G095, G096, or CCI 1PZ21HPD4, IPZ2HQBR, 1PZZ2HQBS, or  Record in the Canadian Organ Replacement Registry indicating receipt of hemodialysis or peritoneal dialysis |
| eGFR (estimated glomerular filtration rate) | Ontario Laboratories Information System | - LOINC for Scr: 14682-9; equation: eGFR = 142 x min(Scr/κ,1)^α^ x max(Scr/κ, 1)^-1.2^ x 0.9938^Age^ x 1.012 [if female]   Where Scr=serum creatinine value (in mg/dL; if in µmol/L, use 0.0113 conversion factor), κ=0.7 for females, 0.9 for males, α=-0.241 for females and -0.302 for males, min indicates the minimum of Scr/κ and 1, and max indicates the maximum of Scr/κ and 1 |
| Hospital Frailty Risk Score | Discharge Abstract Database | Lookback 5 years, using algorithm by Gilbert T et al (Lancet. 2018;391:1775-1782) |
| Excessive Alcohol Use | Discharge Abstract Database  Same Day Surgery Database  National Ambulatory Care Reporting System | ICD-10-CA F10, E52, G62.1, I42.6, K29.2, K70.0, K70.3, K70.9, T51, Z50.2, Z71.4, Z72.1 |
| Stroke or Transient Ischemic Attack | Discharge Abstract Database | ICD-10-CA I63 (excluding I63.6 cerebral infarction due to central venous thrombosis), I64, H34.1, G45.x (excluding G45.4 transient global amnesia), H34.0, or ICD 9-434, 436, 362.3, 435 |
| Liver Dysfunction | Discharge Abstract Database  Same Day Surgery Database  National Ambulatory Care Reporting System | ICD-10-CA I850, I859, I864, I982, K703, K704, K711, K717, K721, K729, K746, K765, K766, K767 |
| Hemoglobin | Ontario Laboratories Information System | The most recent test result in the 1 year prior to and including the date of the index natriuretic peptide test.  LOINC = 718-7, 20509-6 |
| Sodium | Ontario Laboratories Information System | The most recent test result in the 1 year prior to and including the date of the index natriuretic peptide test. LOINC = 2951-2 |
| **Outcome** | **Data Source** | **Definition** |
| Readmission for heart failure | Discharge Abstract Database | Readmission with a primary diagnosis of heart failure within 1 years of the index natriuretic peptide test  ICD-10-CA I50  Exclude admissions flagged as ‘elective’ or where length of stay ≤ 1 day |
| Death – all-cause | Discharge Abstract Database  Registered Persons Database | Fact of death within 1 year of the index natriuretic peptide test    Death date –index natriuretic peptide test date <= 365 days |
| Death – cardiovascular-related | Discharge Abstract Database  Office of the Registrar General - Deaths  Registered Persons Database | Cardiovascular-related cause of death within 1 year of index natriuretic peptide test  Death date – index natriuretic peptide test date <= 365 days and  ICD-10 code = (I00-I79) |

ICD: International Classification of Diseases, CCI: Canadian classification of health interventions, LOINC: Logical Observation Identifiers Names and Code

Supplemental Table 2: Adjusted 1-year rates and 95% Confidence Intervals of clinical outcomes by natriuretic peptide concentration, stratified by sex

| **1-Year All-Cause Mortality** | | |
| --- | --- | --- |
| Natriuretic Peptide (pg/ml) | Male | Female |
| BNP: 400 | 24.23 (24.12, 24.35) | 19.33 (19.2, 19.4) |
| NT-proBNP: 900 | 16.74 (16.23, 17.31) | 13.41 (12.94, 13.82) |
| NT-proBNP: 1800 | 17.82 (17.33, 18.41) | 14.34 (13.82, 14.83) |
| **1-Year Cardiovascular Mortality** | | |
| Natriuretic Peptide (pg/ml | Male | Female |
| BNP: 400 | 3.70 (3.68, 3.71) | 3.16 (3.15, 3.18) |
| NT-proBNP: 2000 | 3.68 (3.66, 3.71) | 2.74 (2.72, 2.76) |
| **1-Year Heart Failure Hospitalization** | | |
| Natriuretic Peptide (pg/ml | Male | Female |
| BNP: 400 | 3.77 (3.74, 3.80) | 3.63 (3.60, 3.66) |
| NT-proBNP: 2000 | 3.67 (3.61, 3.74) | 3.53 (3.47, 3.60) |
